# Dynamic lactate dehydrogenase/lymphocyte ratio and C-reactive protein as predictors of mortality after plasma exchange in ANCA-associated vasculitis: A retrospective case control study

**DOI:** 10.1097/MD.0000000000050314

**Published:** 2026-08-21

**Authors:** Haydar Cagatay Yuksel, Sukriye Miray Kilincer Bozgul, Caner Acar, Gulcin Celebi, Figen Yargucu Zihni, Ajda Gunes, Burcu Barutcuoglu, Devrim Bozkurt

**Affiliations:** a Division of Medical Oncology, Department of Internal Medicine, Ege University Medical Faculty, Izmir, Turkey; b Department of Internal Medicine, Ege University Medical Faculty, Izmir, Turkey; c Division of Rheumatology, Department of Internal Medicine, Ege University Medical Faculty, Izmir, Turkey; d Division of Haematology, Department of Internal Medicine, Ege University Medical Faculty, Izmir, Turkey; e Department of Clinical Biochemistry, Ege University Medical Faculty, Izmir, Turkey.

**Keywords:** biomarkers, C-reactive protein, lactate dehydrogenase, lymphocytes, plasmapheresis, prognosis, vasculitis

## Abstract

The efficacy of plasma exchange (PLEX) in anti-neutrophil cytoplasmic antibody–associated vasculitis (AAV) remains controversial. Early identification of patients unlikely to benefit from PLEX could optimize immunosuppressive strategies. We investigated whether dynamic changes in the lactate dehydrogenase-to-lymphocyte ratio and C-reactive protein (CRP) levels predict in-hospital mortality among critically ill AAV patients undergoing PLEX. In this retrospective, single-center cohort study, 33 adult AAV patients treated with ≥3 PLEX sessions between January 2010 and June 2021 were included. Biochemical parameters were measured immediately before the first and after the final PLEX session. Univariate comparisons between survivors and non-survivors employed Mann–Whitney *U* and chi-square tests as appropriate. A classification and regression tree (CART) model was constructed, following variable selection, variance inflation factor, and recursive feature elimination, to identify key mortality predictors. Model performance was assessed by the area under the curve (AUC) and cross-validation. Median age was 55 years (range 20–83); 57.6% were male. In-hospital mortality was 39.4% (n = 13). Non-survivors exhibited significantly greater post-PLEX increases in LDH/Ly (median 1.1 vs 0.3; *P* < .001) and higher final CRP levels (median 10.2 vs 2.8 mg/L; *P* = .008). The CART model identified post-PLEX LDH-lymphocyte ratio >0.55 as the primary discriminator for mortality, with secondary stratification by CRP > 0.67 mg/dL. The model achieved AUCs of 0.89 (training) and 0.71 (test). Dynamic assessment of LDH-lymphocyte ratio and CRP after PLEX may serve as early, readily available predictors of mortality in critically ill AAV patients. These findings warrant validation in larger, prospective cohorts to refine patient selection for PLEX and tailor adjunctive therapies.

## 1. Introduction

Primary systemic vasculitis causes inflammation of the blood vessels, resulting in occlusive, stenotic, or aneurysmal changes and leading to ischemic or hemorrhagic events.^[1]^ The typical features of granulomatous polyangiitis (GPA) include nasal crusting, stuffiness, and epistaxis; scleritis; upper respiratory tract involvement; and, in the context of active urinary sediment, often kidney involvement. Patients with microscopic polyangiitis (MPA) are typically older and present with more severe kidney disease than those with GPA.^[2]^

In the basic pathophysiology of anti-neutrophil cytoplasmic antibody (ANCA)-associated vasculitis (AAV), antibodies directed against proteinase 3 (PR3) or myeloperoxidase (MPO) play a key role. It has been demonstrated that ANCA activates both neutrophils and monocytes. Incubating MPO-ANCA or PR3-ANCA with normal human neutrophils primed by proinflammatory stimuli, such as tumor necrosis factor-α, bacterial lipopolysaccharide, or complement component 5a, induces a respiratory burst, thereby producing toxic oxygen-free radicals and causing degranulation. This results in the release of numerous destructive enzymes that damage and kill endothelial cells.^[3]^ In addition, during the granulomatous processes predominant in GPA, the inflammatory response involves both CD8^+^ and CD4^+^ T cells as well as Th17 cells, with elevated levels of IL-17 and IL-23 also detected in this context.^[4]^

Inflammation management in patients with AAV typically involves a two-step approach: remission induction and maintenance. Here, induction therapy combines high-dose corticosteroids with an immunosuppressive agent capable of providing long-term suppression, such as cyclophosphamide or rituximab. Plasma exchange (PLEX) can be added to this regimen, although doing so remains controversial. Initially tested during the MEPEX (Methylprednisolone versus Plasma Exchange) trial and later examined during the PEXIVAS study, PLEX has demonstrated benefits in patients with severe renal failure and diffuse alveolar hemorrhage.^[5,6]^ Hence, the 2019 American Society for Apheresis (ASFA) guidelines recommended the use of PLEX as a category I therapy.^[7]^ However, criticisms emerged regarding the limited sample size of the PEXIVAS study, which potentially hindered the demonstration of PLEX’s benefits.^[8]^ Following an expansion of the patient cohort and reevaluation of the data, no significant advantage of PLEX could be established.^[6]^ Furthermore, a recent meta-analysis found no evidence supporting the efficacy of PLEX in reducing mortality, adverse events, or complications.^[9]^ Conversely, a meta-analysis conducted by Walsh et al, who participated in the PEXIVAS study, indicated the benefit of PLEX in preventing progression to end-stage renal disease during a 1-year follow-up period.^[10]^ Still, reflecting these mixed findings, the 2023 ASFA guidelines downgraded PLEX to a category III therapy.^[11]^

In this study, we aimed to identify a specific patient subgroup likely to derive survival benefit from plasma exchange (PLEX). Previous studies have suggested that baseline biochemical parameters at treatment initiation are insufficient to predict this benefit accurately. The uniqueness of our study lies in its systematic evaluation of dynamic biochemical measurements as potential predictors of clinical response to PLEX and associated mortality risk. Thus, this research provides an analytical basis for the personalized application of plasma exchange in clinical practice.

## 2. Materials and methods

### 2.1. Patients

This retrospective single-center study included patients who were diagnosed with AAV and who underwent treatment at the Intensive Care Unit of Ege University’s Faculty of Medicine between January 2010 and June 2021. Data were extracted from patients’ electronic medical records. The inclusion criteria for the study were as follows: over 18 years of age, a confirmed diagnosis of vasculitis based on immunological markers or biopsy, at least 1 life-threatening manifestation^[12]^, and having undergone a minimum of 3 sessions of PLEX. All patients with a life-threatening manifestation of AAV, such as advanced renal failure or severe respiratory abnormalities, who required PLEX for the removal of preformed antibodies until an adequate immunosuppressive effect was achieved, were included in this study.

A total of 74 patients with ANCA-positive vasculitis were initially enrolled in this study. Among them, 31 patients who did not undergo PLEX, 9 patients who were hospitalized for less than 3 days, and 1 patient who underwent only 2 sessions of PLEX were excluded from the study. Hence, a total of 33 patients whose medical records prior to undergoing PLEX were available were ultimately included in this study and divided into 2 groups in terms of mortality during hospitalization. The study adhered to good clinical practice guidelines and the ethical principles outlined in the Declaration of Helsinki. Approval for the study was granted by the Institutional Ethical Review Board of Ege University’s Faculty of Medicine (N:21.9.1 T/1 28.09.2021). Due to the study’s retrospective nature, the need for informed consent from patients was waived.

### 2.2. Treatment protocol

Patients admitted to the intensive care unit were treated according to the same institutional remission induction protocol. The protocol consisted of intravenous cyclophosphamide at a dose of 1 g, followed by high-dose corticosteroid therapy with intravenous methylprednisolone 500 mg/d for 3 consecutive days. Thereafter, corticosteroids were tapered day by day according to clinical status, organ involvement, infection risk, and the treating physician’s judgment. In addition, patients meeting the study inclusion criteria underwent daily PLEX via a central venous catheter with 5% albumin as the replacement fluid.

### 2.3. Statistical analysis

The statistical analyses in this study were conducted using jamovi (version 2.3.28, the jamovi project, 2023, https://www.jamovi.org), JASP (version 0.18.3, Jeffreys’ Amazing Statistics Program, 2024, https://jasp-stats.org), Minitab® 21.4.2 (64-bit; ©2023 Minitab, LLC. All rights reserved), and R version 4.4.0. Moreover, the statistical analyses in R were performed using the caret, Boruta, rpart, and lime packages. The statistical significance level was set at *P* ≤ .05.

Descriptive statistics were used to summarize the data obtained in this study. Continuous variables were presented as the median, minimum, and maximum values, depending on the distribution. Categorical variables were summarized as counts and percentages. The normality of numerical variables was assessed using appropriate tests and visual tools based on the sample size and data characteristics. The Shapiro–Wilk test was preferred for comparisons with small sample sizes (n < 50). Additionally, histograms and Q-Q plots were used to evaluate the assumption of normality. The biochemical laboratory values were analyzed in 2 groups: samples collected at the start of PLEX (labeled with “1”) and samples collected after the final plasmapheresis session (labeled with “2”). For categorical variables, Pearson chi-square, Fisher exact and Fisher–Freeman–Halton tests were used. The Mann–Whitney *U* test was used for non-normally distributed data. Indices and their calculation methods are provided in Supplementary Material 1, Supplemental Digital Content 1.

A classification and regression tree (CART) analysis, which is a machine-learning method, was used to determine the mortality risk in this study. Prior to the analysis, the Boruta, variance inflation factor (VIF), and recursive feature elimination (RFE) methods were used to select the most significant variables for the CART model. More specifically, the Boruta algorithm was used to select the most important variables within the model, while the VIF analysis was used to investigate any multicollinearity issues. The RFE method identified variables that optimized the model performance. Following these preliminary analyses, the data were divided into a training set and a test set. The performance of the final CART model (mortality ~ albumin + globulin + C-reactive protein [CRP] + direct bilirubin-2 + neutrophil/lymphocyte ratio [NLR] + systemic inflammatory index [SII] + lactate dehydrogenase [LDH]/lymphocyte ratio + prognostic nutritional index [PNI]) was evaluated using metrics such as the mean log-likelihood, area under the receiver operating characteristic curve (AUC), lift, and misclassification cost. The accuracy of the model was assessed via cross-validation. Subsequently, a local interpretable model-agnostic explanations (LIME) analysis was conducted to explain the model’s decisions and determine the variables’ significance.

## 3. Results

The median age at hospitalization among the 33 patients included in this study was 55 years (Range 20–83). Among the patients, 57.6% were male (n = 19) and 42.4% were female (n = 14). The median duration of hospital stay was 17 days (Range 4–43). All the patients had renal involvement, while pulmonary involvement was observed in 25 patients (69.7%) and hematological conditions were noted in 14 patients (42.4%). Moreover, gastrointestinal system involvement was seen in 2 patients (6.1%). The 5-factor score distribution was as follows: 4 patients (12.1%) with a score of zero, 6 patients (18.2%) with a score of 1, 22 patients (66.7%) with a score of 2, and 1 patient (3.0%) with a score of 3. The patients’ diagnoses included MPA in 9 patients (27.3%) and GPA in 24 patients (72.7%). All the patients included in this study were treated with an induction therapy regimen consisting of cyclophosphamide, PLEX, and high-dose steroids (see Table S1, Supplemental Digital Content 2). When assessing the disease activity using the Birmingham Vasculitis Activity Score (BVAS), the median score was found to be 15 (range: 10–23). A total of 16 patients (48.5%) experienced an infection during their intensive care unit (ICU) stay. Furthermore, pneumonia occurred in 6 patients (18.2%), urinary tract infection was observed in eleven patients (33.3%), and gastroenteritis was identified in 1 patient (3.0%). Notably, 2 patients (6.1%) experienced catheter-related blood infection. Positive blood cultures were found in 8 patients (24.2%). During PLEX, 23 patients (69.7%) underwent hemodialysis, 16 patients (48.5%) received erythrocyte suspension (ES), and 16 patients (48.5%) received intravenous immune globulin. The median number of days spent undergoing PLEX was 4 (range 3–9). Seven patients (21.2%) required noninvasive mechanical ventilation, 14 patients (42.4%) were intubated, and 13 patients (39.4%) received inotropic therapy. The median Sequential Organ Failure Assessment score was 8 (range: 5–13), while the median Acute Physiology and Chronic Health Evaluation II score was 28 (range: 24–38). The causes of death among non-survivors were reviewed from the available clinical records and are summarized in Table S2, Supplemental Digital Content 3. Overall, death was primarily attributed to AAV-related organ failure in 9 patients, septic shock in 3 patients, and an undetermined cause in 1 patient. No patient had documented infection before or during the early PLEX assessment period. Infectious complications, including pneumonia, urinary tract infection, gastroenteritis, and bloodstream infection, were documented later during ICU follow-up in some patients.

Non-survivors were significantly older than survivors (*P* = .005). The number of patients with pneumonia was significantly higher in the mortality group (*P* = .025), and the mortality group also had a significantly higher number of patients who received ES during PLEX (*P* = .023; Table 1).

**Table 1:** Clinical and demographic characteristics and pairwise comparisons in terms of mortality in patients with vasculitis.

	Overall (n = 33)	Mortality		*P*-values
		Yes (n = 13)	No (n = 20)	
Diagnosis age†	55.0 [20.0–83.0]	64.0 [36.0–83.0]	54.0 [20.0–64.0]	**.005** *
Gender‡				
Male	19 (57.6)	9 (69.2)	10 (50.0)	.464**
Female	14 (42.4)	4 (30.8)	10 (50.0)	
Hospital stay duration†	17.0 [4.0–43.0]	15.0 [4.0–43.0]	18.0 [4.0–42.0]	.580*
Renal involvement, yes‡	33 (100.0)	13 (100.0)	20 (100.0)	.99
Lung involvement, yes‡	25 (75.8)	11 (84.6)	14 (70.0)	.431**
Hematological condition, yes‡	14 (42.4)	8 (61.5)	6 (30.0)	.152**
Gastrointestianal system, yes‡	2 (6.1)	1 (7.7)	1 (5.0)	.999**
Five factor score‡				
0	4 (12.1)	1 (7.7)	3 (15.0)	.472**
1	6 (18.2)	4 (30.8)	2 (10.0)	
2	22 (66.7)	8 (61.5)	14 (70.0)	
3	1 (3.0)	0 (0.0)	1 (5.0)	
BVAS	15 (10–23)	16.3 (12–23)	15.5 (10–23)	.867
Diagnosis‡				
mPAN	9 (27.2)	4 (30.8)	5 (25.0)	.642**
GPA	24 (72.7)	9 (69.2)	15 (75.0)	
Antibody‡				
c ANCA	25 (72.7)	9 (69.2)	16 (80.0)	.642**
p ANCA	9 (27.2)	4 (30.8)	4 (20.0)	
C3†	111.0 [29.0–171.0]	111.0 [29.0–148.0]	112.5 [43.0–171.0]	.682*
C4†	27.0 [5.0–55.0]	22.0 [8.0–55.0]	27.0 [5.0–50.0]	.720*
Infection, yes‡	16 (48.5)	8 (61.5)	8 (40.0)	.394**
Pneumonia, yes‡	6 (18.2)	5 (38.5)	1 (5.0)	**.025** **
Urinary tract, yes‡	11 (33.3)	4 (30.8)	7 (35.0)	.999**
Gastrointestianal, yes‡	1 (3.0)	1 (7.7)	0 (0.0)	.394**
Catheter-associated bloodstream infection, yes‡	2 (6.1)	2 (15.4)	0 (0.0)	.148**
Positive blood culture, yes‡	8 (24.2)	5 (38.5)	3 (15.0)	.213**
During plasmapheresis				
Hemodialysis, yes‡	23 (69.7)	11 (84.6)	12 (60.0)	.245**
Erythrocyte replacement, yes‡	16 (48.5)	10 (76.9)	6 (30.0)	**.023** **
IVIG, yes‡	16 (48.5)	5 (38.5)	11 (55.0)	.567**
Plasmapheresis days†	5.0 [3.0–9.0]	4.0 [3.0–9.0]	5.0 [3.0–7.0]	.627*
NIMV (before PLEX), yes‡	6 (18.8)	4 (33.3)	2 (10.0)	.102**
Intubation (before PLEX), yes‡	1 (3.0)	1 (7.7)	0 (0.0)	.208**
Inotropic support (before PLEX), yes‡	6 (18.2)	4 (30,8)	2 (10.0)	.131**
NIMV (during hospitalization), yes‡	7 (21.2)	5 (38.5)	2 (10.0)	.084**
Intubation (during hospitalization), yes‡	14 (42.4)	12 (92.3)	2 (10.0)	**<.001** **
Inotropic support (during hospitalization), yes‡	13 (39.4)	12 (92.3)	1 (5.0)	**<.001** **
SOFA score†	8 [5.0–13.0]	8.0 [5.0–13.0]	8.0 [6.0–13.0]	.910*
APACHE II score	28 [24.0–38.0]	29 [25.0–38.0]	27.5 [24.0–36.0]	.193

Bold values indicate statistically significant results, defined as *P* < .05.

ANCA = anti-neutrophil cytoplasmic antibody, APACHE II = Acute Physiology and Chronic Health Evaluation II, BVAS = Birmingham Vascultis Activity İndex, GPA = granulomatosis with polyangiitis, IVIG = intravenous immunoglobulin, mPAN = microscopic polyangiitis, NIVM = non-invasive mechanical ventilation, SOFA = Sequential Organ Failure Assessment.

† Median [min–max].

‡ n (%).

* Mann–Whitney *U* test.

** Pearson Chi-Square, Fisher Exact/Fisher Freeman Halton test.

In addition, comparison of the patients with and without mortality status revealed significant differences in the aspartate transferase (AST)-2 (*P* = .042), alanine transaminase (ALT)-2 (*P* = .042), total bilirubin-2 (*P* = .039), direct bilirubin-2 (*P* = .010), and globulin-2 (*P* = .009) levels, with these values being significantly higher in the patients with mortality. Conversely, the albumin-2 (*P* = .008) levels were significantly lower in the mortality group (Table 2). In terms of the immunological parameters, the CRP-2 (*P* = .008), LDH-2 (*P* = .027), and NLR-2 (*P* = .014) values were significantly higher in the patients with mortality, whereas the lymphocyte-2 (*P* = .001) levels were significantly lower in the non-survivor patients (Table S3, Supplemental Digital Content 4).

**Table 2:** Comparison of inflammatory and prognostic indices between patients with different mortality outcomes.

	Overall	Mortality		*P*-values*
		Yes (n = 13)	No (n = 20)	
Score 1.1 – Systemic Immune-Inflammation Index 1	314.9 [180.0–1782.4]	211.7 [18–1782.4]	376.9 [10.1–1091.1]	.207
Score 1.2 – Systemic Immune-Inflammation Index 2	233. [63.1–1532.6]	482.6 [63.1–1532,6]	185.7 [174,4–551.8]	.068
Score 2.1 – LDH/albumin ratio 1	88.3 [45.5–364.2]	133.0 [45.5–287.6]	87.8 [46.1–364.2]	.676
Score 2.2 – LDH/albumin ratio 2	97.0 [47.0–2855.7]	117.1 [69.7–2855.7]	77.9 [47.0–266.0]	**.008**
Score 3.1 – LDH/lymphocyte ratio 1	0.4 [0.1–2.4]	0.6 [0.2–2.4]	0.3 [0.1–1.7]	.071
Score 3.2 – LDH/lymphocyte ratio 2	0.5 [0.0–15.9]	1.1 [0.3–15.9]	0.3 [0.0–2.9]	**<.001**
Score 4.1 – lymphocyte/CRP ratio 1	6.3 [0.05–98.9]	2.93 [0.05–41.1]	7.6 [0.2–98.9]	.137
Score 4.2 – lymphocyte/CRP ratio 2	30.16 [0.01–24.5]	5.71 [0.01–12.8]	91.4 [2.7–245.3]	**<.001**
Score 5.1 – albumin/NLR ratio 1	0.2 [0.1–29.2]	0.2 [0.1–29.2]	0.2 [0.1–0.5]	.366
Score 5.2 – albumin/NLR ratio 2	0.3 [0.0–57.0]	0.1 [0.0–57.0]	0.3 [0.1–1.2]	**.013**
Score 6.1 – Prognostic Nutritional Index 1	35.2 [15.5–48.5]	32.2 [15.5–41.0]	35.3 [30.8–48.5]	.052
Score 6.2 – Prognostic Nutritional Index 2	35.0 [26.0–57.0]	32.2 [26.0–42.6]	39.3 [30.6–57.0]	**<.001**

The Δ (Delta) symbol represents change, while Δ % represents the percentage change between 2 measurements.

Bold values indicate statistically significant results, defined as *P* < .05.

* Mann–Whitney *U* test.

When comparing the tested indices between the patients with and without mortality status, significant differences were observed in the LDH/albumin ratio-2 (*P* = .008), LDH/lymphocyte ratio-2 (*P* < .001), lymphocyte/CRP ratio-2 (*P* < .001), albumin/NLR ratio-2 (*P* = .013), and PNI-2 (*P* < .001), with these values being significantly higher in the patients with mortality (Table 2).

**Figure 1.**
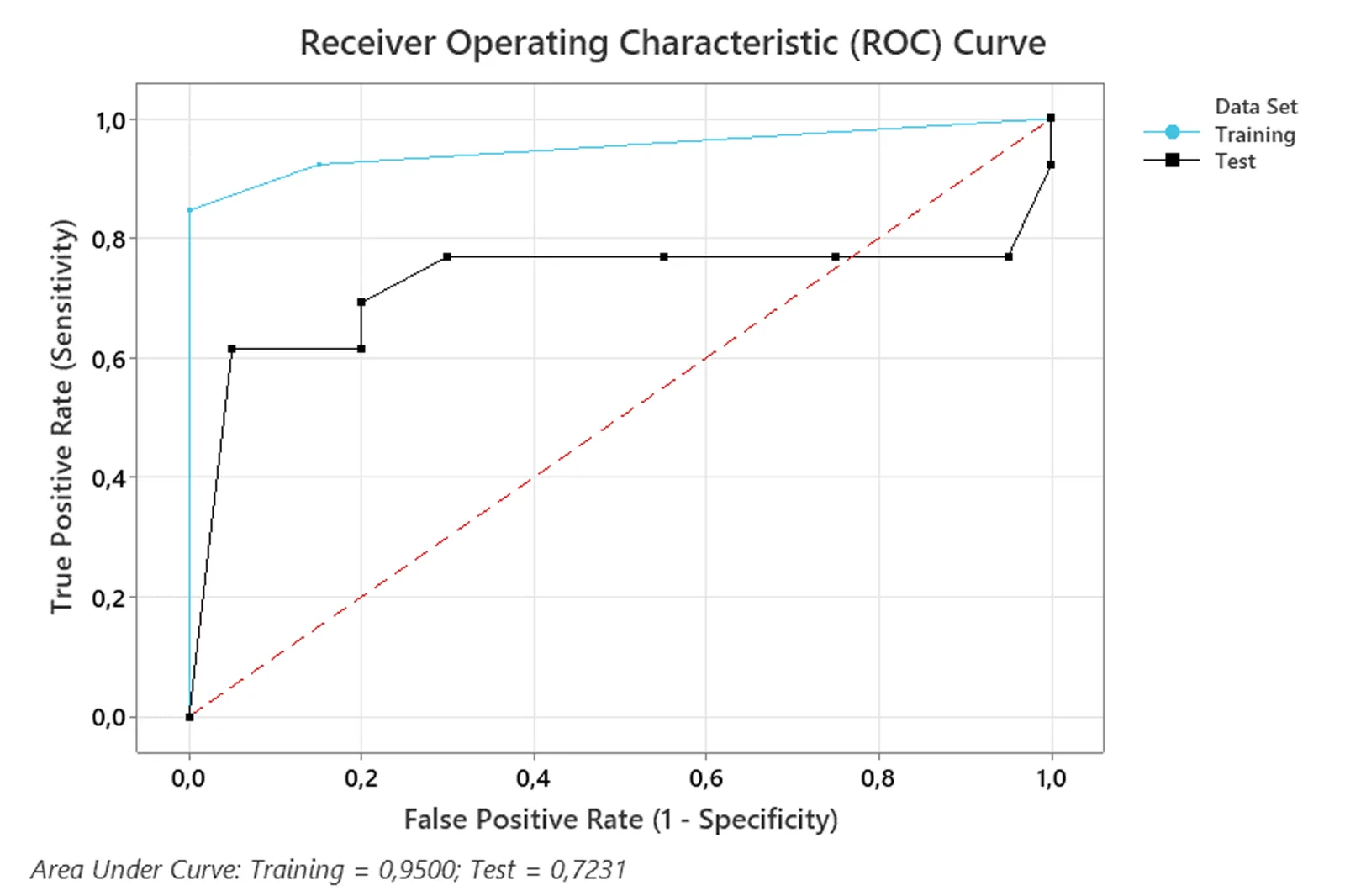
Receiver operating characteristic curve of the CART model for mortality prediction. The receiver operating characteristic (ROC) curves show the discriminative performance of the CART model in the training and test datasets. The area under the curve was 0.950 for the training dataset and 0.723 for the test dataset. The red dashed diagonal line represents the reference line for no discrimination. CART = classification and regression tree, ROC = receiver operating characteristic.

The mean negative log-likelihood for the training and test datasets was 0.3445 and 0.5664, respectively. The AUC was 0.8865 (95% CI [0.2649, 1]) for the training dataset and 0.7115 (95% CI [0.5041, 0.9190]) for the test dataset (Fig. 1). The lift value of the model was 2.0308 for the training dataset and 1.2692 for the test dataset. Moreover, the misclassification cost was 0.2269 for the training dataset and 0.4308 for the test dataset. Hence, the model demonstrated robust performance on the training dataset; however, its performance declined on the test dataset (Table 3).

**Table 3:** Performance statistics of the CART model for mortality prediction for training and test dataset.

Statistics	Training	Test
Average–loglikelihood	0.345	0.566
Area under ROC curve	0.887	0.712
95% CI	(0.265; 1.01)	(0.504; 0.919)
Lift	20.308	12.692
Misclassification cost	0.227	0.431

CART = classification and regression tree, CI = confidence interval, ROC = receiver operating characteristic.

* The table summarizes the performance metrics of the CART model for predicting mortality in the training and test datasets, showing the model’s strong performance in the training dataset with a decline in performance in the test dataset.

In terms of the training dataset, the sensitivity (true positive rate), specificity (true negative rate), type I error rate (false positive rate), and type II error rate (false negative rate) of the model were 92.3%, 85.0%, 15.0%, and 7.7%, respectively. With regard to the test dataset, the sensitivity of the model was 76.9%, the specificity was 80.0%, the type I error rate was 20.0%, and the type II error rate was 23.1%. These results show that the model exhibited high sensitivity and specificity values in relation to the training dataset, although its performance decreased in the test dataset (Table 4).

**Table 4:** Classification performance statistics of the CART model for mortality prediction for training and test dataset.

Statistics	Predicted class (training)	Predicted class (test)
	Training (%)	Test (%)
True positive rate (sensitivity or power)	92.3	76.9
False positive rate (type I error)	15	20
False negative rate (type II error)	7.7	23.1
True negative rate (specificity)	85	80

The table displays the classification performance metrics of the CART model for predicting mortality in the training and test datasets, indicating higher sensitivity and specificity in the training dataset compared to the test dataset.

CART = classification and regression tree.

**Figure 2.**
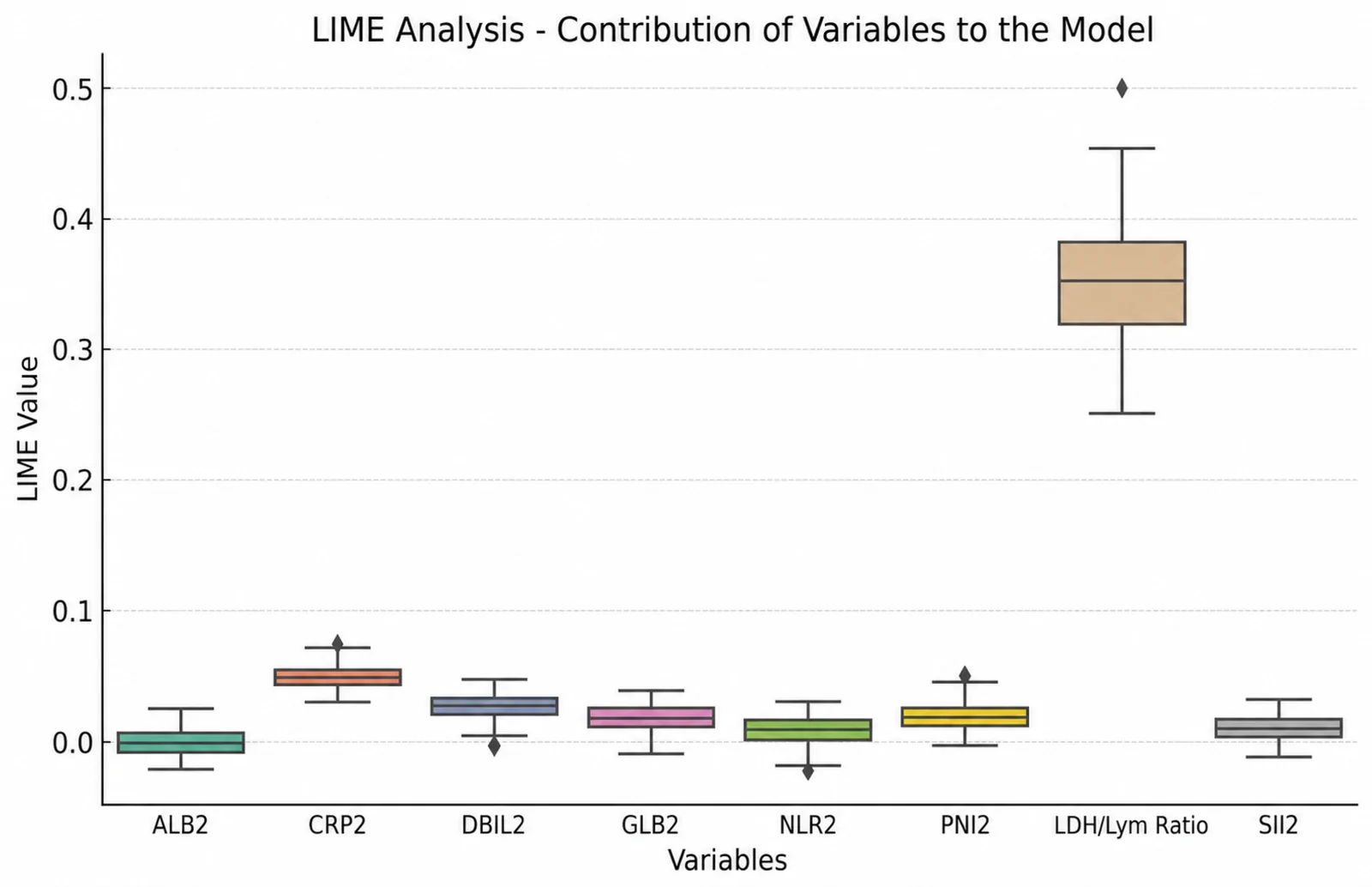
LIME-based variable contribution analysis of the CART model. Higher LIME values indicate a greater contribution to the mortality prediction model. The LDH/lymphocyte ratio after final PLEX had the highest contribution. The suffix “2” denotes values measured after the final PLEX session. CART = classification and regression tree, CRP = C-reactive protein, DBIL = direct bilirubin, GLB = globulin, LDH/Lym ratio = lactate dehydrogenase/lymphocyte ratio, LIME = local interpretable model-agnostic explanations, NLR = neutrophil/lymphocyte ratio, PNI = prognostic nutritional index, SII = systemic immune-inflammation index.

A Local Interpretable Model-agnostic Explanations analysis was conducted to evaluate the contribution of each variable within the model. The LDH/lymphocyte ratio was identified as the most critical variable when it came to improving the model’s performance. Here, an increase in the LDH/lymphocyte ratio values was associated with a significant elevation in the mortality risk. Additionally, variables such as the PNI, GLB, NLR, and CRP levels contributed to the model, albeit their effects were more limited. An increase in the PNI values resulted in a reduction in the mortality risk, although the effects were limited. The ALB and SII variables made a negligible contribution to the model (Fig. 2).

**Figure 3.**
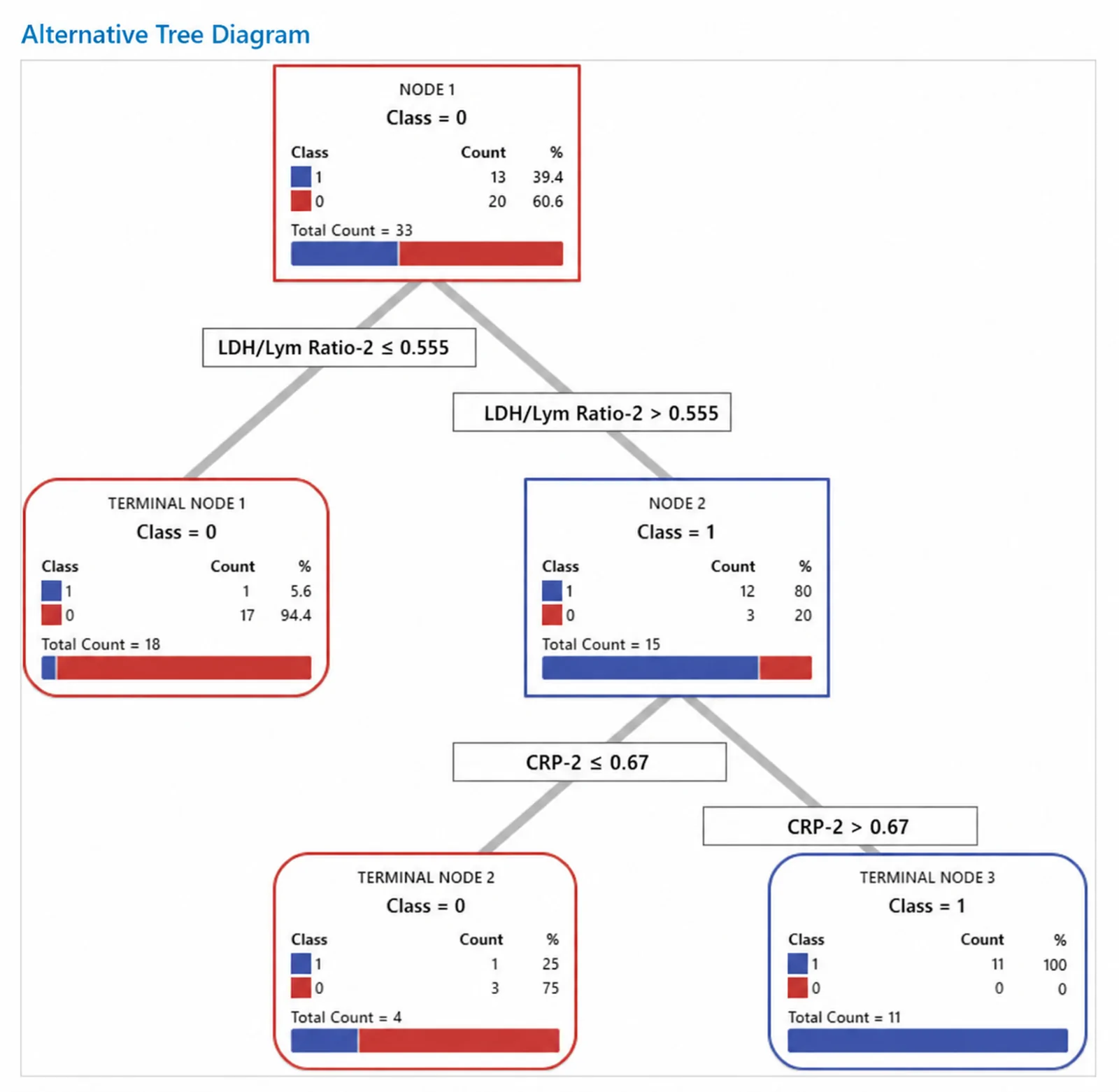
CART-based decision tree for mortality prediction after PLEX in patients with AAV. The decision tree stratifies patients according to the LDH/lymphocyte ratio after final PLEX and CRP after final PLEX. Class 0 indicates survival, and class 1 indicates in-hospital mortality. The first split was based on the post-PLEX LDH/lymphocyte ratio, with values >0.553 identifying patients at higher mortality risk. Among these patients, post-PLEX CRP further stratified risk, with CRP > 0.67 identifying the terminal node with the highest mortality probability. The bar plots within each node show the distribution of survival and mortality classes. AAV = anti-neutrophil cytoplasmic antibody-associated vasculitis, CART = classification and regression tree, CRP = C-reactive protein, LDH/Lym ratio = lactate dehydrogenase/lymphocyte ratio, PLEX = plasma exchange.

The decision tree, which was generated via the CART analysis, illustrated the critical junctures in the variables employed to anticipate mortality and the efficacy of such determinations in the classification (Fig. 3). At the root node of the tree structure, the LDH/lymphocyte ratio was identified as the most significant discriminator variable. When the LDH/lymphocyte ratio value was less than or equal to 0.553, the model directed the classification decision to Terminal Node 1, where the probability of survival (class 0) was 94.4%, with 17 out of 18 observations classified as class 0 and 1 as class 1 (death). If the LDH/lymphocyte ratio value exceeded 0.553, the model was directed to Node 2, where the CRP variable was designated as the secondary parameter. When the CRP-2 value was less than or equal to 0.67, the classification was directed to Terminal Node 2, where the probability of survival (class 0) was 75%, with 3 out of 4 observations classified as class 0 and 1 as class 1. When the CRP value exceeded 6.7, the model was directed to Terminal Node 3, where the probability of death (class 1) was set to 100%, and all 11 observations were classified as class 1.

The LDH/lymphocyte ratio, which was identified as the best predictor variable, was found to be higher in the group of patients who experienced mortality. After the first PLEX session, the difference became statistically significant (*P* = .017). Similarly, the LDH/lymphocyte ratio retained its significance in the evaluations conducted after subsequent PLEX sessions.

## 4. Discussion

AAV is a systemic disease characterized by life-threatening flares. However, only a limited number of studies have investigated the outcomes of AAV in critically ill patients who require intensive care, while studies that specifically evaluated the efficacy of PLEX in such patients are even scarcer. This study examined the clinicopathological features and biochemical parameters that may predict the response to PLEX in patients with AAV who are admitted to ICUs.

In patients with vasculitis who require intensive care, the in-hospital mortality rates range from 15.5% to 58.8%.^[13]^ Among the most common reasons for admission are renal failure and the need for hemodialysis.^[14]^ Respiratory complications, such as massive hemoptysis due to pulmonary involvement, represent another major cause of ICU admission.^[15]^ Moreover, central nervous system involvement, severe cytopenia, and gastrointestinal system involvement may necessitate intensive care.^[16]^ Contrary to findings reported in the literature, renal involvement was more prevalent than pulmonary involvement in this study. This discrepancy may be attributed to the frequent evaluation of data from internal medicine ICUs. In patients with life-threatening conditions, the primary treatment option is remission induction therapy, which consists of high-dose corticosteroids combined with an immunosuppressive agent such as cyclophosphamide or rituximab.^[12]^

As a systemic inflammatory disease, AAV is characterized by increased inflammatory markers and a deterioration in parameters that are indicative of organ dysfunction at the time of admission. AAV primarily causes tissue damage mediated by antibody-driven innate immunity, with neutrophils playing a central role.^[17]^ Additionally, lymphopenia is considered to reflect the burden of inflammation, as seen in conditions such as rheumatoid arthritis, Crohn disease, systemic lupus erythematosus and vasculitis. Lymphopenia at the time of diagnosis in patients with AAV, particularly patients with renal involvement, has been shown to correlate with disease severity and renal prognosis.^[18]^ However, some studies have reported a negative correlation between the lymphocyte count and the recurrence rate, with lymphopenia being associated with a lower rate of recurrence.^[19]^ In this study, while the patients’ lymphocyte counts at ICU admission did not show a statistically significant difference in terms of the mortality rate, a rising trend in lymphocyte levels was observed in those patients who responded to PLEX. By contrast, the lymphocyte counts continued to decline in the mortality group. As observed in previous studies, low lymphocyte levels may be indicative of effective clearance of circulating inflammatory markers, potentially explaining the reduced recurrence rates seen in these patients.

LDH is one of the key markers indicating cellular death.^[20]^ Necrosis of the vascular endothelium mediated by neutrophil-driven immune responses forms the cornerstone of AAV’s pathophysiology.^[3]^ Thus, LDH appears to be a logical marker for defining disease activity as an indicator of this necrosis,^[20]^ although there is limited research on this topic. In the present study, the LDH levels measured on the third day of PLEX were observed to continue rising in the patients with mortality, whereas a decline was noted in the patients without mortality.

CRP is a protein synthesized in the liver in response to IL-6, and it is a component of the innate immune system. Its primary function is to bind to complement proteins, enhancing the opsonization and neutralization of antigens.^[21]^ In AAV, the activation of innate immunity results in elevated CRP levels, which trigger inflammation. The high CRP levels in AAV have been associated with complement deposition in the kidneys.^[22]^ Additionally, when evaluated alongside albumin as part of the CRP/albumin ratio score, CRP has been identified as a predictor of mortality.^[19]^ In this study, the CRP levels showed a decline in both groups; however, a significant reduction was only observed in the patients without mortality. Moreover, the median CRP level after PLEX in the patients with mortality was approximately 10 times higher than the normal range.

The rationale for evaluating the LDH/lymphocyte ratio was based on its ability to integrate 2 biologically distinct but clinically complementary processes. LDH primarily reflects cellular injury, tissue hypoxia, endothelial damage, and metabolic stress, all of which may occur during severe vasculitic organ involvement and critical illness.^[20,23]^ In contrast, lymphocyte count reflects host immune reserve and may decrease in the setting of systemic inflammation, severe physiological stress, immunosuppressive therapy, and infection-related immune dysregulation.^[24,25]^ Therefore, a high LDH/lymphocyte ratio may represent the coexistence of ongoing tissue injury and impaired immune recovery more effectively than either LDH or lymphocyte count alone. In our model, CRP provided an additional inflammatory dimension, reflecting persistent acute-phase activation after PLEX.^[19]^ Thus, the combination of LDH/lymphocyte ratio and CRP may capture different aspects of post-PLEX clinical risk: tissue injury and metabolic stress, impaired immune reserve, and unresolved systemic inflammation. Although composite inflammatory indices such as the neutrophil/lymphocyte ratio, systemic immune-inflammation index, and CRP/albumin ratio have previously been evaluated in AAV, the LDH/lymphocyte ratio has not been well established in this setting.^[19]^ Therefore, our findings should be interpreted as exploratory and hypothesis-generating, requiring validation in larger prospective AAV cohorts.

A relevant issue is the potential overlap between active vasculitic inflammation and infectious complications in critically ill patients with AAV. Both life-threatening vasculitic activity requiring ICU admission and infectious complications are important determinants of mortality.^[26]^ Therefore, the LDH/lymphocyte ratio and CRP may theoretically be influenced by both active AAV and infection-related systemic inflammation.^[27]^ However, in the present study, these markers were evaluated during the early PLEX period, and no patient had documented infection before or during this early assessment period. In contrast, several infectious complications were documented later during ICU follow-up. This temporal distinction suggests that the early elevation of the LDH/lymphocyte ratio and persistent CRP after PLEX should not be interpreted simply as a consequence of late hospital-acquired infection. Rather, these markers may reflect unresolved systemic inflammatory burden, vasculitic tissue injury, metabolic stress, and impaired immune recovery after PLEX. Accordingly, the LDH/lymphocyte ratio and CRP should be considered early prognostic indicators of post-PLEX clinical risk rather than AAV-specific disease activity markers. Nevertheless, later nosocomial infections, particularly pneumonia, contributed to mortality and were more frequent among non-survivors, highlighting the importance of close infection surveillance and early management during ICU follow-up.

While the value of PLEX in AAV has been deprioritized following the PEXIVAS study, it may be premature to conclude that no patient groups benefit from this therapy. Indeed, factors such as the use of non-albumin solutions for PLEX, short treatment durations, and potential investigator biases raise concerns about the reliability of the PEXIVAS study’s findings.^[28]^ Reflecting this uncertainty, the updated European Alliance of Associations for Rheumatology guidelines from 2024 recommend a patient-specific approach to evaluating the efficacy of PLEX.^[12]^ Similarly, the ASFA guidelines classify PLEX as a category III therapy.^[11]^ Nevertheless, unanswered questions remain. It is evident that the immune responses of patients undergoing PLEX, as well as their subsequent treatment outcomes, are not uniform. Studies based on kidney biopsies have suggested that renal involvement may serve as a predictor of the response to PLEX.^[29,30]^ However, kidney biopsies are not feasible in every patient, particularly in emergency situations, where simpler and more accessible methods are required.

In this study, it was observed that, among the 2 populations with similar baseline biochemical and clinicopathological characteristics (except for age), the changes during follow-up were more effective in determining the benefit of PLEX than the initial parameters. As novel markers, the LDH/lymphocyte ratio and CRP levels can serve as potential predictors of the response to PLEX, with the trends in these parameters during follow-up offering valuable insights into the treatment efficacy. In this study, a decrease in LDH levels and an increase in lymphocyte counts were observed in patients who responded to PLEX, whereas the opposite patterns were noted in those who did not respond.

However, the findings of this study also raise new questions. For instance, the role of PLEX, while beneficial in certain contexts, requires further exploration so that its benefits and risks can be better balanced. The potential of specific biochemical markers, such as the LDH/lymphocyte ratio and CRP level, as prognostic tools also warrants further investigation. This study had several limitations. It was a single-center study with a small sample size and a low number of mortality events. Additionally, due to being a retrospective study, it was not possible to control for all the potential confounding factors. For instance, critical information such as the patients’ medication history, disease history, nutritional status and comorbidities was not consistently documented in their medical records. Although no patient had documented infection before or during the early PLEX assessment period, later ICU-related infectious complications occurred in some patients and contributed to mortality. Therefore, we could not fully disentangle the relative contributions of active AAV, possible occult early infection, and subsequent hospital-acquired complications to mortality. Since this study included only critically ill ICU patients, the findings may not be generalizable to the broader AAV population. Finally, long-term follow-up outcomes were not assessed.

## 5. Conclusion

This study highlights the critical role of biochemical parameters and clinical interventions in predicting mortality among patients with AAV. The findings demonstrate that the age at diagnosis, pneumonia, and need for erythrocyte transfusion are all predictors of mortality. The elevated levels of AST, ALT, bilirubin, globulin, CRP, LDH, and NLR observed in the mortality group underscore the importance of these markers in assessing disease severity and prognosis. Conversely, the lower albumin and lymphocyte counts in the mortality group further emphasize their role as indicators of poor outcomes.

This study also elucidates the efficacy of and risks associated with PLEX in managing AAV. While PLEX is a crucial intervention for severe cases, the present results suggest that its benefits must be weighed against the potential complications, particularly in patients who require additional supportive measures. The CART model developed in this study effectively identifies key predictors of mortality, including the LDH/lymphocyte ratio, PNI, globulin, NLR, and CRP levels, providing a valuable tool that clinicians can use to stratify risk and tailor treatment strategies accordingly.

This research contributes to the growing body of evidence concerning the prognostic factors associated with AAV and highlights the need for early identification and targeted management of high-risk patients. The integration of biochemical markers into routine clinical practice could enhance the accuracy of prognosis and improve patient outcomes.

## Acknowledgments

We express our deepest gratitude to the patients and our families for their support and to the circumstances that guided us.

## Author contributions

**Conceptualization:** Haydar Cagatay Yuksel, Sukriye Miray Kilincer Bozgul, Devrim Bozkurt.

**Data curation:** Haydar Cagatay Yuksel, Caner Acar, Gulcin Celebi.

**Formal analysis:** Haydar Cagatay Yuksel, Caner Acar, Ajda Gunes.

**Investigation:** Haydar Cagatay Yuksel.

**Methodology:** Figen Yargucu Zihni.

**Supervision:** Burcu Barutcuoglu, Devrim Bozkurt.

**Writing – original draft:** Haydar Cagatay Yuksel, Sukriye Miray Kilincer Bozgul, Devrim Bozkurt.

**Writing – review & editing:** Haydar Cagatay Yuksel, Sukriye Miray Kilincer Bozgul, Devrim Bozkurt.








