# Supplementary material for: Dynamic lactate dehydrogenase/lymphocyte ratio and C-reactive protein as predictors of mortality after plasma exchange in ANCA-associated vasculitis: A retrospective case control study
Source: Medicine (Baltimore). 2026 Aug 21;105(34):e50314. doi: 10.1097/MD.0000000000050314 (PMC13502825; doi:10.1097/MD.0000000000050314)
Supplement: Supplementary file 1 [file medi-105-e50314-s001.docx]

**Supplementary Material 1: Index Calculation Formulas**

**Neutrophil-to-Lymphocyte Ratio (NLR):** Neutrophil (10³ x μL) / Lymphocyte (10³ x μL)

**Platelet-to-Lymphocyte Ratio (PLR):** Platelet (10³ x μL) / Lymphocyte (10³ x μL)

**Systemic Inflammatory Index (SII):** Neutrophil (10⁶ x μL) × Platelet (10⁶ x μL) / Lymphocyte (10⁶ x μL)

**Lactate Dehydrogenase-to-Lymphocyte Ratio (LDH/L):** Lactate Dehydrogenase (U/L) / Lymphocyte (10³ x μL)

**Prognostic Nutritional Index (PNI):** Albumin level (g/L) + 0.005 × Lymphocyte (10³ x μL)
