# Supplementary material for: Dynamic lactate dehydrogenase/lymphocyte ratio and C-reactive protein as predictors of mortality after plasma exchange in ANCA-associated vasculitis: A retrospective case control study
Source: Medicine (Baltimore). 2026 Aug 21;105(34):e50314. doi: 10.1097/MD.0000000000050314 (PMC13502825; doi:10.1097/MD.0000000000050314)
Supplement: Supplementary file 2 [file medi-105-e50314-s002.docx]

**Supplementary Table 1 :** Patients characteersitc before plasmapheresis

| **Patient No** | **Sex** | **Age** | **Diag** | **FFS** | **BVAS** | **Plasmapheresis** | **Plasmapheresis Session** | **APACHE** | **HD** | **IVIG** | **Mortality** | **Organ inv** |
| --- | --- | --- | --- | --- | --- | --- | --- | --- | --- | --- | --- | --- |
| ***1*** | *F* | *64* | *GPA* | 2 | 19 | Pulmonary | 3 | 31 | + | - | *ex* | *K,L,H* |
| ***2*** | *M* | *58* | *GPA* | 2 | 21 | Pulmonary | 6 | 33 | - | - | *ex* | *K,L,* |
| ***3*** | *M* | *47* | *GPA* | 1 | 14 | Renal | 3 | 28 | + | + | *ex* | *K,H* |
| ***4*** | *M* | *83* | *mPAN* | 2 | 23 | Renal | 4 | 37 | + | - | *ex* | *K,L* |
| ***5*** | *F* | *64* | *mPAN* | 2 | 15 | Pulmoneral | 5 | 25 | + | - | *ex* | *K,L,G* |
| ***6*** | *M* | *61* | *GPA* | 1 | 13 | Pulmoneral | 3 | 28 | + | - | *ex* | *K,L* |
| ***7*** | *M* | *74* | *mPAN* | 2 | 16 | Pulmonary | 3 | 28 | + | - | *ex* | *K,L,H* |
| ***8*** | *M* | *68* | *GPA* | 1 | 15 | Pulmonary | 6 | 29 | + | + | *ex* | *K,L* |
| ***9*** | *M* | *55* | *GPA* | 2 | 13 | Renal | 6 | 27 | + | + | *ex* | *K,H* |
| ***10*** | *M* | *79* | *GPA* | 1 | 14 | Pulmonary | 3 | 25 | - | + | *ex* | *K,L,* |
| ***11*** | *F* | *67* | *GPA* | 1 | 16 | Renal | 5 | 31 | + | + | *ex* | *K,H* |
| ***12*** | *F* | *36* | *GPA* | 2 | 14 | Pulmonary | 9 | 29 | + | - | *ex* | *K,L,* |
| ***13*** | *M* | *55* | *mPAN* | 2 | 12 | Renal | 4 | 27 | + | - | *ex* | *K,L,H* |
| ***14*** | *F* | *20* | *GPA* | 2 | 23 | Renal | 5 | 38 | + | + | - | *K,L,H* |
| ***15*** | *F* | *44* | *mPAN* | 2 | 12 | Renal | 3 | 27 | + | - | - | *K,H* |
| ***16*** | *M* | *54* | *GPA* | 2 | 19 | Renal | 5 | 33 | + | + | - | *K,L* |
| ***17*** | *M* | *34* | *GPA* | 0 | 21 | Pulmonary | 3 | 36 | - | - | - | *K,L* |
| ***18*** | *M* | *45* | *GPA* | 2 | 14 | Renal | 7 | 25 | + | + | - | *K,L,H* |
| ***19*** | *M* | *63* | *GPA* | 2 | 16 | Renal | 7 | 27 | + | + | - | K,H |
| ***20*** | *M* | *46* | *GPA* | 2 | 23 | Renal | 3 | 33 | + | - | - | K,G |
| ***21*** | *F* | *54* | *mPAN* | 2 | 14 | Renal | 4 | 25 | + | + | - | *K,L* |
| ***22*** | *F* | *55* | *GPA* | 2 | 16 | Pulmonary | 5 | 27 | - | - | - | *K,L,H* |
| ***23*** | *M* | *26* | *GPA* | 2 | 14 | Renal | 5 | 25 | + | - | - | K,L |
| ***24*** | *M* | *53* | *GPA* | 0 | 12 | Pulmonary | 5 | 27 | - | - | - | K,H |
| ***25*** | *F* | *54* | *GPA* | 0 | 23 | Pulmonary | 5 | 33 | - | - | - | K,L |
| ***26*** | *F* | *64* | *GPA* | 2 | 12 | Pulmonary | 5 | 25 | - | + | - | K,L |
| ***27*** | *F* | *63* | *mPAN* | 1 | 10 | Renal | 5 | 24 | + | + | - | K,H |
| ***28*** | *M* | *56* | *GPA* | 2 | 16 | Renal | 5 | 28 | - | + | - | K,L |
| ***29*** | *M* | *59* | *GPA* | 3 | 17 | Pulmonary | 5 | 30 | - | - | - | K,L |
| ***30*** | *F* | *37* | *GPA* | 2 | 15 | Renal | 5 | 31 | + | + | - | K |
| ***31*** | *M* | *61* | *mPAN* | 2 | 19 | Renal | 5 | 33 | + | - | - | K,L |
| ***32*** | *F* | *20* | *GPA* | 1 | 10 | Renal | 5 | 24 | + | + | - | K |
| ***33*** | *F* | *57* | *mPAN* | 2 | 16 | Renal | 3 | 28 | + | + | - | K,L |

Abr: F: Female, M: Male , GPA: Granulomatosis with Polyangiitis , MPA: Microscopic Polyangiitis, FFS: Five Factor Score, HD: Hemodialysis, IVIG: Intravenous Immunoglobulin, K: Kidney, L: Lung, H: Hematological, G: Gastrointestinal System
