# Supplementary material for: Dynamic lactate dehydrogenase/lymphocyte ratio and C-reactive protein as predictors of mortality after plasma exchange in ANCA-associated vasculitis: A retrospective case control study
Source: Medicine (Baltimore). 2026 Aug 21;105(34):e50314. doi: 10.1097/MD.0000000000050314 (PMC13502825; doi:10.1097/MD.0000000000050314)
Supplement: Supplementary file 3 [file medi-105-e50314-s003.docx]

**Supplementary Table 2.** Causes of death among non-survivors

| **Patient no.** | **Age, years** | **AAV diagnosis** | **Organ involment** | **Infection documented before/early PLEX** | **Infection during ICU follow-up** | **Primary cause of death** |
| --- | --- | --- | --- | --- | --- | --- |
| 1 | *64* | *GPA* | *K,L,H* | No | None | AAV-related organ failure |
| 2 | *58* | *GPA* | *K,L,* | No | Pneumonia | Septic shock |
| 3 | *47* | *GPA* | *K,H* | No | None | AAV-related organ failure |
| 4 | *83* | *MPA* | *K,L* | No | UTI | AAV-related organ failure |
| 5 | *64* | *MPA* | *K,L,G* | No | Pneumonia/BSI | Septic shock |
| 6 | *61* | *GPA* | *K,L* | No | UTI | AAV-related organ failure |
| 7 | *74* | *MPA* | *K,L,H* | No | Gastroenteritis | AAV-related organ failure |
| 8 | *68* | *GPA* | *K,L* | No | None | AAV-related organ failure |
| 9 | *55* | *GPA* | *K,H* | No | Pneumonia/BSI | Septic shock |
| 10 | *79* | *GPA* | *K,L,* | No | None | Undetermined |
| 11 | *67* | *GPA* | *K,H* | No | UTI | AAV-related organ failure |
| 12 | *36* | *GPA* | *K,L,* | No | None | AAV-related organ failure |
| 13 | *55* | *MPA* | *K,L,H* | No | UTI | AAV-related organ failure |

Abr: , GPA: Granulomatosis with Polyangiitis , MPA: Microscopic Polyangiitis,K: Kidney, L: Lung, H: Haematological, UTI: Urinary Tract Infection, BSA: Bloodstream Infection
