# Supplementary material for: Dynamic lactate dehydrogenase/lymphocyte ratio and C-reactive protein as predictors of mortality after plasma exchange in ANCA-associated vasculitis: A retrospective case control study
Source: Medicine (Baltimore). 2026 Aug 21;105(34):e50314. doi: 10.1097/MD.0000000000050314 (PMC13502825; doi:10.1097/MD.0000000000050314)
Supplement: Supplementary file 4 [file medi-105-e50314-s004.docx]

**Supplementary Table 3 :** Comparison of Biochemical Values Between Patients with Different Mortality Outcomes

|  |  | **Mortality** | |  |
| --- | --- | --- | --- | --- |
| **Variables** | **Overall** | **Yes (n=13)** | **No (n=20)** | **p-values*** |
| **AST 1(U/L)** | 18.0 [5.0 – 1105.0] | 18.0 [8.0 – 165.0] | 18.5 [5.0 – 1105.0] | 0.632 |
| **AST 2 (U/L)** | 16.0 [5.0 – 4708.0] | 21.0 [10.0 – 4708.0] | 13.0 [5.0 – 39.0] | **0.042** |
| **ALT 1 (U/L)** | 13.0 [6.0 – 1055.0] | 13.0 [7.0 – 127.0] | 14.0 [6.0 – 1055.0] | 0.796 |
| **ALT 2 (U/L)** | 16.0 [5.0 – 1840.0] | 21.0 [12.0 – 1840.0] | 15.0 [5.0 – 72.0] | **0.042** |
| **Total Bilirubin 1 (mg/dl)** | 0.5 [0.1 – 2.1] | 0.6 [0.3 – 2.1] | 0.4 [0.1 – 2.0] | 0.320 |
| **Total Bilirubin 2 (mg/dl)** | 0.7 [0.1 – 4.2] | 0.9 [0.2 – 4.2] | 0.6 [0.1 – 1.3] | **0.039** |
| **Direct Bilirubin 1 (mg/dl)** | 0.2 [0.1 – 1.9] | 0.4 [0.1 – 1.9] | 0.2 [0.1 – 1.5] | 0.073 |
| **Direct Bilirubin 2 (mg/dl)** | 0.3 [0.1 – 3.4] | 0.4 [0.1 – 3.4] | 0.2 [0.1 – 0.6] | **0.010** |
| **Albumin 1 (gL)** | 3.0 [1.4 – 3.9] | 2.9 [1.4 – 3.9] | 3.0 [2.1 – 3.9] | 0.177 |
| **Albumin 2 (g/L)** | 3.3 [2.4 – 4.1] | 3.0 [2.4 – 3.5] | 3.3 [3.0 – 4.1] | **0.008** |
| **Globulin 1 (mg/dl)** | 3.1 [1.9 – 5.6] | 3.2 [2.2 – 4.0] | 3.1 [1.9 – 5.6] | 0.605 |
| **Globulin 2 (mg/dl)** | 2.5 [1.6 – 5.8] | 2.9 [1.6 – 4.4] | 2.3 [1.7 – 5.8] | **0.032** |
| **Creatinine 1 (mg/dl)** | 4.7 [0.6 – 9.0] | 4.4 [0.6 – 8.2] | 5.2 [0.7 – 9.0] | 0.372 |
| **Creatinine 2 (mg/dl)** | 3.8 [0.5 – 11.3] | 3.0 [1.1 – 6.2] | 4.0 [0.5 – 11.3] | 0.501 |
| **CRP 1 (mg/L)** | 135 [8 – 309.0] | 135 [8 – 309.0] | 107 [10 – 183.0] | 0.353 |
| **CRP 2 (mg/L)** | 27 [1 – 324.0] | 50 [4 – 324.0] | 18 [1 – 21.7] | **0.008** |
| **LDH 1 (U/L)** | 283.0 [150.0 – 1311.0] | 277.0 [150.0 – 834.0] | 285.5 [166.0 – 1311.0] | 0.985 |
| **LDH 2 (U/L)** | 318.0 [155.0 – 7996.0] | 410.0 [209.0 – 7996.0] | 265.5 [155.0 – 963.0] | **0.027** |
| **WBC 1 (10^3^/µ)** | 13.8[0.3- 26.4] | 11.1 [0.3 – 23.9] | 14.1 [5530.0 – 26490.0] | 0.102 |
| **WBC 2 (10^3^/µ)** | 12.2 [0.2 – 40.6] | 13.2 [0.2 – 40.6] | 11.3 [6.0 – 20.6] | 0.598 |
| **Neutrophil 1(10^3^/µ)** | 11.5 [30.0 – 23.1] | 9970.0 [30.0 – 21.7] | 12.4 [3.9 – 23.1] | 0.118 |
| **Neutrophil 2 (10^3^/µ)** | 11.3 [1.0 – 38.9] | 12.1 [1.0 – 38.9] | 9.7 [5.3 – 15.2] | 0.281 |
| **Lymphocyte 1 (10^3^/µ)** | 0.8 [0.1 – 2.5] | 0.5 [0.1 – 1.5] | 0.9 [0.4 – 2.5] | 0.055 |
| **Lymphocyte 2 (10^3^/µ)** | 0.7 [0.02– 3.4] | 0.3 [0.02 – 1.5] | 0.9 [0.1 – 3.4] | **0.001** |
| **Eosinophil 1 (10^3^/µ)** | 0.1 [0.0 – 1.9] | 0.0 [0.0 – 1.1] | 0.1 [0.0 – 1.9] | 0.530 |
| **Eosinophil 2 (10^3^/µ)** | 0.0 [0.0 – 0.08] | 0.0 [0.0 – 0.04] | 0.05 [0.0 – 0.08] | 0.127 |
| **Hemoglobin 1 (g/dl)** | 8.9 [4.9 – 11.7] | 9.1 [6.2 – 11.6] | 8.8 [4.9 – 11.7] | 0.428 |
| **Hemoglobin 2 (g/dl)** | 8.7 [6.8 – 11.9] | 8.6 [6.8 – 10.2] | 8.9 [7.3 – 11.9] | 0.135 |
| **Platelet 1 (10^3^/µ)** | 264.0 [95.0 – 494.0] | 210.0 [95.0 – 462.0] | 279.0 [148.0 – 494.0] | 0.065 |
| **Platelet 2 (10^3^/µ)** | 162.0 [120.0 – 419.0] | 158.0 [120.0 – 281000.0] | 188.0 [39.0 – 419.0] | 0.179 |
| **Platelet/Lymphocyte Ratio 1** | 339.3 [38.0 – 1613.0] | 441.7 [38.0 – 1613.0] | 305.0 [125.7 – 827.8] | 0.703 |
| **Platelet/Lymphocyte Ratio 2** | 231.8 [31.7 – 6350.0] | 306.2 [63.2 – 6350.0] | 186.0 [31.7 – 486.7] | 0.062 |
| **Neutrophil/Lymphocyte Ratio 1** | 11.9 [0.1 – 66.4] | 14.4 [0.1 – 66.4] | 11.6 [6.8 – 37.0] | 0.768 |
| **Neutrophil/Lymphocyte Ratio 2** | 13.7 [0.0 – 97.0] | 38.0 [0.0 – 97.0] | 12.1 [2.7 – 44.5] | **0.014** |

Data are presented as median [minimum–maximum].
*. Mann–Whitney U test.

Abr: ALT: Alanine aminotransferase AST: Aspartate aminotransferase CRP: C-reactive protein LDH: Lactate Dehydrogenases WBC: white blood cell
